# LINC01419 facilitates hepatocellular carcinoma growth and metastasis through targeting EZH2-regulated RECK

**DOI:** 10.18632/aging.103321

**Published:** 2020-06-10

**Authors:** Gong Zhang, Ximin Chen, Lei Ma, Rui Ding, Lihong Zhao, Feng Ma, Xubin Deng

**Affiliations:** 1 Department of Radiotherapy, People's Hospital of Shanxi Province, Taiyuan, China; 2 Affiliated Cancer Hospital and Institute of Guangzhou Medical University, Guangzhou, China; 3 Department of Gastroenterology, The First Affiliated Hospital of Jinan University, Guangzhou, China

**Keywords:** hepatocellular carcinoma, LINC01419, EZH2, RECK

## Abstract

Long non-coding RNAs (lncRNAs) have been reported to play significant roles in human tumorigenesis, for example, in hepatocellular carcinoma (HCC). This study explored the role of LINC01419, a new lncRNA, in HCC. *In vitro* study revealed that LINC01419 promotes growth and migration of HCC cells. Genes that affected cell proliferation and cell migration were identified using RNA-sequence. Subsequently, it was confirmed that LINC01419 binds to EZH2, leading to histone methylation of the RECK promoter. Interaction between LINC01419 and FUS stabilized EZH2 mRNA thereby enhancing EZH2 expression. Conclusively, the results of this study confirm that LINC01419 may serve as a potential target for HCC diagnosis and treatment.

## INTRODUCTION

Hepatocellular carcinoma (HCC) is one of the most prevalent and fatal human cancers globally. Current HCC treatments include surgical resection, molecular-targeted therapy, and immunotherapy [1]. Advancement in HCC treatment has been made over the past decades, however, the prognosis of HCC patients is still substandard [2]. HCC disease is insidious and patient diagnosis occurs at the advanced stages. There is an urgent need to elucidate the molecular mechanism of HCC and develop new therapeutic targets.

Long non-coding RNAs (lncRNAs) are implicated in diverse cellular processes and disease pathogenesis [3]. LncRNAs exert their functions in multiple ways, for example, they serve as signal mediators, molecular decoys and they scaffold or enhances transcription [4]. Currently, researchers have identified a lncRNAs that are associated with HCC. Similarly, previous studies have reported that abnormal expression of lncRNAs contributes to HCC progression [5–7]. However, the underlying mechanisms have not been properly determined.

This study aims to investigate the biological role of LINC01419 and the underlying mechanism in HCC.

## RESULTS

### LINC01419 expression level is elevated in HCC tissues and is associated with a malignant phenotype

**Figure 1:**
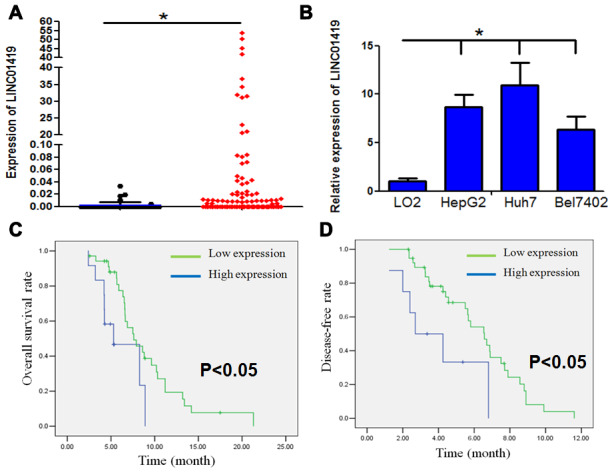
**LINC01419 is highly expressed and associated with malignant phenotypes in HCC.** (**A**) The expression level of LINC01419 in HCC tissues and normal liver tissues in the TCGA cohort has been indicated. (**B**) Showing relative LINC01419 expression in HCC cell lines and normal human liver cell line LO2. (**C**) HCC patients with high LINC01419expression level exhibited a low PFS rate than those with low LINC01419expression level. (**D**) HCC patients with high LINC01419expression levels had a low OS rate than those with low LINC01419expression levels as conformed using Kaplan-Meier assay.

In investigating the role of lncRNAs in HCC progression, the lncRNA expression profile was analyzed in HCC samples and non-HCC liver samples. The samples were obtained from TCGA datasets via circlncRNAnet (http://app.cgu.edu.tw/circlnc/) and TANRIC (http://ibl.mdanderson.org/tanric/_design/basic/index.html). Several abnormal lncRNAs were detected and showed significant statistical differences. This study focused on LINC01419 because it showed the most significant difference (Figure 1A, Supplementary Figure 1A).

The LINC01419 expression level was higher in HCC cell lines when compared to LO2 (normal liver cell line), (Figure 1B). To confirm whether LINC01419is a non-coding RNA, online bioinformatics analysis showed that LINC01419 had no coding capability (CPAT: Coding-Potential Assessment Tool) http://lilab.research.bcm.edu/cpat/index.php), (Supplementary Figure 1B). *In vitro* translation experiments proved that LINC01419 does not have coding ability (Supplementary Figure 1C). Analysis by subcellular fractionation and real-time PCR showed that LINC01419 was mainly localized within the cytoplasm (Supplementary Figure 1D).

The relationship between LINC01419 expression and the clinicopathological characteristics of patients has been highlighted in Table 1. The elevated LINC01419 expression level was significantly associated with clinicopathological characteristics including tumor size, lymph node metastasis, and advanced clinical stage. However, other clinicopathological characteristics, for example, age, gender, and HBV infection were not correlated with LINC01419 expression level. Kaplan-Meier analysis showed that HCC patients with higher LINC01419 expression levels had shorter overall survival and disease-free time than those with lower LINC01419 expression level (Figure 1C and 1D, P<0.05).

**Table 1:** Associations between lncRNALINC01419 expression and patients’ clinicopathological features.

**Variable**	**No. of patients**	**LINC01419 low expression**	**LINC01419 high expression**	**P value**
**Age**				
<60	20	10	10	0.9
≥60	27	13	14	
**Gende**r				
Male	29	14	15	0.908
Female	18	9	9	
**Tumor size**				
<5cm	25	18	7	0.001
≥5cm	22	5	17	
**Lymph node involvement**				
Absent(pN0)	25	16	9	0.028
Present(pN+)	22	7	15	
**TNM stage**				
I-II	31	20	11	0.003
III-IV	16	3	13	
**HBV infection**				
Yes	21	9	12	0.454
NO	26	14	12	

### *In vitro* LINC01419 silencing inhibits proliferation and migration of HCC cells

**Figure 2:**
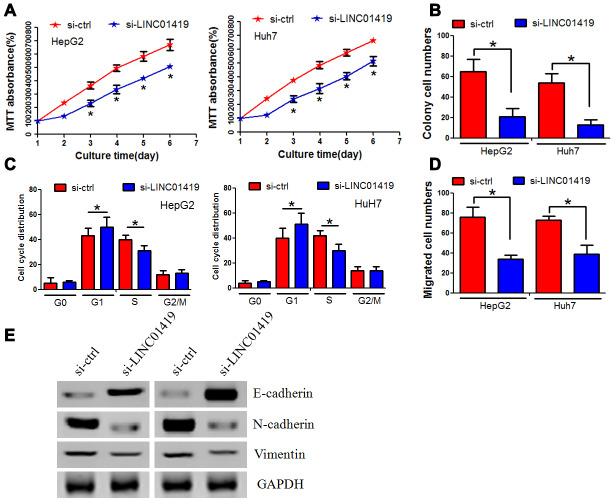
**Inhibiting LINC01419 decreases HCC cell proliferation and invasion.** (**A**) Cell viability examination using MTT assay. (**B**) Showing impaired colony-forming ability in LINC01419-silenced cells. (**C**) Flow cytometry assay used to examine cell cycle distribution. (**D**) Examining HCC cell migration ability using transwell assay. (**E**) Protein levels of E-cadherin, N-cadherin, and Vimentin examination by western blot assay.

In evaluating the biological function of LINC01419 in HCC, siRNA was used to knockout the endogenous expression of LINC01419 (Supplementary Figure 2A). MTT assay indicated that LINC01419 silencing significantly inhibited the growth of HepG2 and Huh7 cells (Figure 2A). Through colony formation analysis, LINC01419 knockout significantly reduced the colony formation ability of liver cancer cells (Figure 2B, Supplementary Figure 2B). Flow cytometry assay was used to determine whether LINC01419 affected cell cycle distribution. LINC01419 downregulation resulted in increased cell frequency in the G1 phase whereas, cell frequency was decreased in the S phase (Figure 2C, Supplementary Figure 2C). Subsequently, the Boyden test was used to determine whether LINC01419 affected the invasion of HCC cells. It was reported that LINC01419 inhibition reduced HCC cell invasion (Figure 2D, Supplementary Figure 2D). Interestingly, when LINC01419 was inhibited, change in epithelial-mesenchymal transformation-related markers was observed. In sh-LINC01419 cells, E-cadherin expression was increased, whereas, the N-cadherin and Vimentin expression were decreased (Figure 2E).

In summary, these results implicated that LINC01419 promoted *in vitro* proliferation and invasion of HCC cells.

### LINC01419 silences RECK epigenetically by binding to EZH2

**Figure 3:**
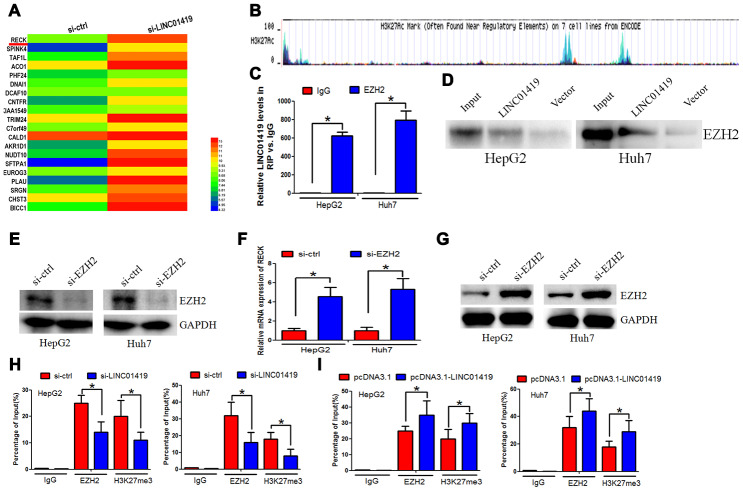
**LINC01419 silences RECK epigenetically by binding to EZH2.** (**A**) The different gene transcripts expression between si-ctrl cells and si- LINC01419 cells, demonstrated by hierarchical cluster. (**B**) The promoter regions of RECK showing EZH2 transcriptional sites, as indicated by UCSC. (**C**) LINC01419 interaction with EZH2, as revealed by the RIP experiments. (**D**) Desthiobiotinylation-LINC01419 bound EZH2 in HCC cells, as indicated by the pull-down assays.(E)Shows EZH2 down-regulation by si-RNA in HCC cells, and the knockdown efficiency examination using western blot assay. (**F**) qPCR assay examination of the mRNA expression level of RECK. (**G**) The western blot analysis of the RECK protein expression level. (**H**) Showing EZH2 and H3K27me3 enriched in the RECK promoter regions as indicated by CHIP assay. (**I**) Sowing increased EZH2 and H3K27me3levelsafter LINC01419 overexpression in HCC cell.

RNA transcriptome sequencing was used to identify the potential target genes correlated with LINC01419.Series of genes were either up-regulated or down-regulated (fold change≥4-fold) after the LINC01419 knockout. Genetic ontology analysis was performed to determine the most significant biological behavioral pathways for protein binding, RNA binding, and DNA binding (Supplementary Figure 1E). The KEGG pathway analysis revealed that different genes mainly participated in cancer (Supplementary Figure 1F). A significant increase in RECK was detected after the LINC01419 knockdown (Figure 3A).

EZH2 was reported to epigenetically inhibit transcription of downstream genes. Hypermethylation of the promoter contributed to RECK downregulation in cancer, this was verified in the UCSC database (http://genome.ucsc.edu/), (Figure 3B). It was, therefore, postulated that RECK may be regulated throughEZH2 transcription. Reports from recent studies indicate that lncRNAs can co-regulate gene silencing with chromatin-modifying enzymes, for example, EZH2. Based on this, LINC01419 may regulate the expression of RECK by binding to EZH2.There was endogenous LINC01419 precipitation in the anti-EZH2 antibody components in comparison to the non-specific IgG control group. This suggested a possible interaction between EZH2 and LINC01419 (Figure 3C). RNA pull-down analysis showed that LINC01419 RNA, rather than the vector, especially retrieved EZH2 from the HepG2 nuclear extract, further confirmed the binding of EZH2 to LINC01419 (Figure 3D). To determine whether EZH2 modulates RECK transcription through H3K27me3, EZH2 expression knockdown was performed (Figure 3E). Elevated levels of RECK proteins and mRNA was reported (Figure 3F, 3G). Subsequently, we determined the effect of reduced or increased levels ofLINC01419 on EZH2 and H3K27me3 enrichment in the RECK promoter region. Through CHIP and qPCR analysis, it was reported that LINC01419 knockout inhibited EZH2 binding and H3K27me3 levels of RECK promoter (Figure 3H). Contrarily, when LINC01419 was overexpressed in HepG2 and Huh7 cells, EZH2 and H3K27me3 binding levels were elevated in the RECK promoter (Figure 3I). The above results confirmed that LINC01419 catalyzes H3K27me3 in the RECK promoter region by binding to EZH2.LINC01419 thus partially inhibits the apparent expression of RECK and promotes growth and migration of HCC cells.

### LINC01419 recruits FUS and stabilizes EZH2 mRNA

**Figure 4:**
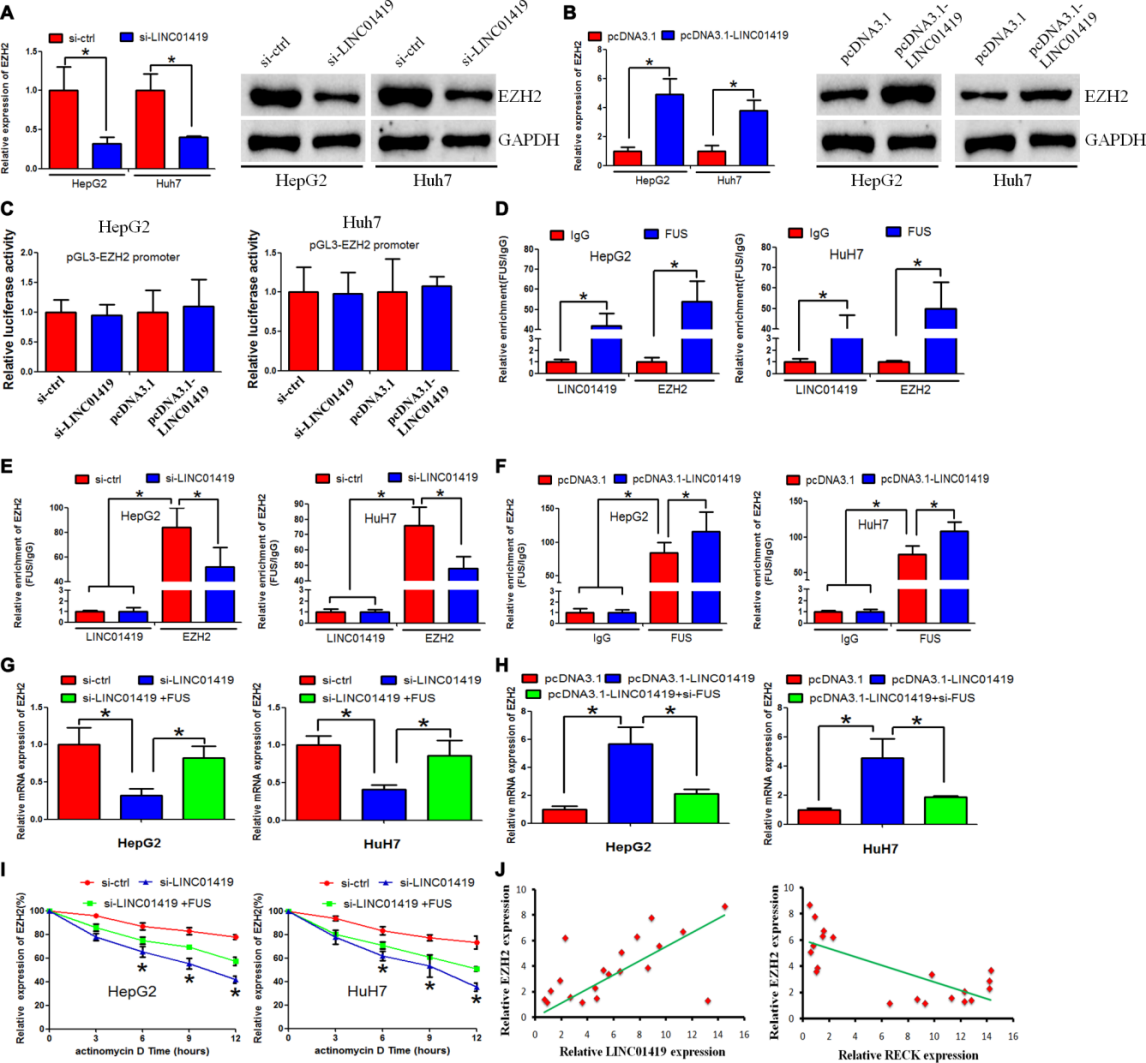
**LINC01419 stabilizes EZH2 mRNA by recruiting FUS.** (**A**, **B**) Showing RT-PCR and western blot assays used to examine EZH2 expression levels in HCC cells when LINC01419 was inhibited or overexpressed, respectively. (**C**) Luciferase reporter assay showing that LINC01419 did not affect EZH2 transcription. (**D**) FUS interaction with LINC01419 and EZH2-mRNA as validated by the RIP assay. (**E**) The estimated impact of LINC01419 down-regulation on FUS interaction with EZH2-mRNA using RIP assay. (**F**) The estimated impact of LINC01419 overexpression on FUS–interaction with EZH2 -mRNA using RIP assay. (**G**–**H**) A qRT-PCR assay used to examine the EZH2 expression level. (**I**) The degradation rate of EZH2-mRNA after treatment with actinomycin D. (**J**) Right panel: Correlation between LINC01419 expression level and EZH2 expression level examined by RT-PCR; Left panel: Correlation between RECK expression level and EZH2 expression level examined by RT-PCR.

The mechanism of LINC01419 in modulating EZH2 in HCC was explored. Expression of mRNA and protein levels of EZH2 decreased when LINC01419 was inhibited, whereas, it increased when LINC01419 was overexpressed (Figure 4A and 4B). From this, it was proved that LINC01419 positively regulates EZH2 inHCC. However, down-regulation or up-regulation of LINC01419 significantly did not affect the luciferase activity of the EZH2 promoter, as revealed in HepG2 and Huh7 cells (Figure 4C). Therefore, it was proposed that LINC01419 takes part in the post-transcriptional regulation of EZH2 in HCC. Reports from the literature indicate that RNA-binding protein (RBP) complicit lncRNA-regulated gene expression. The RBP, FUS, was shown to interact with LINC01419 and EZH2-mRNA, this was verified with the starBase 2.0 (http://starbase.sysu.edu.cn/starbase2/index.php). This study determined whether FUS enhanced EZH2 expression inLINC01419. Through RIP analysis, it was reported that LINC01419 and EZH2 were significantly captured by FUS in HepG2 and Huh7 cells (Figure 4D). In addition, FUS bound EZH2-mRNA levels decreased significantly with LINC01419 silencing, whereas, the levels increased with LINC01419 overexpression (Figure 4E and 4F). The role of LINC01419 in inhibiting or stimulating EZH2 expression in HCC cells is partially offset through FUS up-regulation or down-regulation (Figure 4G and 4H). Besides, LINC01419 down-regulation accelerated the degradation rate of EZH2-mRNA.This effect was reversed through forced FUS expression (Figure 4I). These results suggested that LINC01419 regulates EZH2-mRNA stability, and this is mediated by FUS. Furthermore, a positive correlation between LINC01419 expression level and the EZH2 expression level was noted (Figure 4J, left panel). However, a negative correlation existed between the EZH2 expression level and RECK expression level (Figure 4J, right panel).

### Regulatory effect of LINC01419 on RECK and its potential oncogenic function

**Figure 5:**
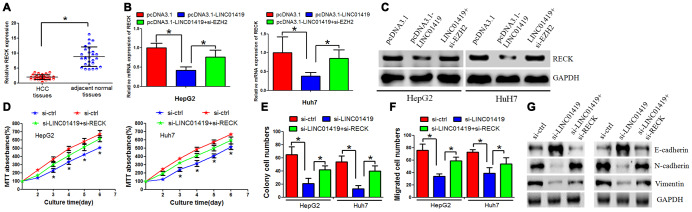
**RECK suppresses HCC cell proliferation and metastasis, and counteracts LINC01419 activity.** (**A**) RT-PCR assay to examine the RECK expression level. (**B**) The mRNA level of RECK as examined by qRT-PCR. (**C**) The RECK protein level as examined by western blot assay. (**D**–**F**) Analysis of cells by MTT assays (**D**), colony formation (**E**), and transwell assays (**F**) (**G**) Western blot assay was performed to examine E-cadherin, N-cadherin and Vimentin expression levels.

This study determined the biological role of RECK in HCC cells. It was reported that the RECK-mRNA expression level decreased in HCC tissues when compared with the adjacent normal tissues (Figure 5A). Subsequently, we investigated the potential role of RECK in mediating LINC01419 function in HCC cells. When LINC01419 was overexpressed in HepG2 and Huh7 cells, decreased expression level of RECK was noted (Figure 5B and 5C). However, RECK inhibition by LINC01419 was reversed at mRNA and protein levels. This occurred after EZH2 down-regulation with si-RNAs (Figure 5B and 5C). A rescue assay was performed by co-transfecting HCC cells with LINC01419 and RECK siRNAs. This explored the underlying effects of LINC01419/RECK on cell growth and invasion. It was revealed that RECK down-regulation partially reversed the effect of LINC01419 down-regulation on growth and migration (Figure 5D–5F). We also overexpressed RECK in HCC cells and found that RCEK decreased cell growth and migration ability (Supplementary Figure 2E–2G). In addition, we found that inhibition of LINC01419 elevated RECK expression level (Supplementary Figure 2H).

### c-jun elevated LINC01419 expression in HCC

**Figure 6:**
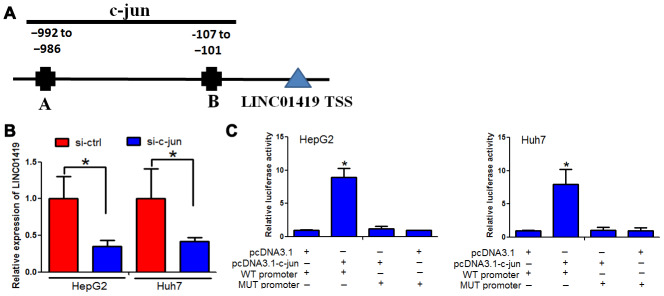
**c-jun elevated LINC01419 expression in HCC.** (**A**) Indicates the promoter regions of LINC01419 with the putative c-jun TFBS. (**B**) RT-PCR assay used to examine LINC01419 expression levels. (**C**) Elevated luciferase activity in wild-type LINC01419 promoter caused by c-jun.

Transcriptional activation causes dysregulation of downstream genes. In this context, it was examined whether LINC01419upregulation is caused by transcriptional activation. Two bioinformatics software (UCSC and PROMO) were used to analyze the 1000bp region, upstream of the LINC01419 transcription initiation site. Two c-jun-binding motifs at-992 to -986 and −107 to −101 were identified in the promoter region, upstream of the LINC01419 transcription initiation site. The two transcription factor-binding sites (TFBSs) were named A and B, respectively (Figure 6A).

Furthermore, we determined whether c-jun modulates LINC01419 expression. c-jun down-regulation by si-RNAs inhibited LINC01419 in HCC cells (Figure 6B). To confirm c-jun binding at the LINC01419 promoter, chromatin immune-precipitation and q-PCR assays were performed. When c-jun was overexpressed, luciferase activity in wild-type LINC01419 promoter increased (Figure 6C). However, c-jun overexpression had no significant effect on luciferase activity when c-jun binding sites on LINC01419 were mutated (Figure 6C). Based on these results, it was suggested that c-jun binding to the LINC01287 promoter positively regulates its expression.

## DISCUSSION

Many reports have recommended lncRNAs as vital regulators of gene expression and cancer development over the past few decades. From our previous studies, for example, we reported that LINC01287 and XIST could function as oncogenic lncRNA in human HCC [5, 7]. LINC01419 was shown to promote tumor progression, this led to substandard prognosis in different types of cancers [8–10]. Elsewhere, a study showed that LINC01419 was significantly overexpressed in HBV-related and HCV-related HCC [11]. However, limited information exists on the biological role of LINC01419 in HCC. This propelled us to perform an in-depth exploration of the underlying LINC01419 mechanisms.

In this study, we determined that the LINC01419 expression level was elevated in HCC. This was verified using lncRNA raw data from TCGA. Our results indicated that LINC01419 inhibition could decrease HCC cell proliferation and migration ability. Moreover, through *in vivo* experiments, LINC01419 downregulation was shown to suppress HCC proliferation. In addition, an *in vivo* study revealed that inhibiting LINC01419 impaired proliferation ability of HCC cells. Despite having reported that LINC01419 functions as an onco-lncRNA in HCC, its relevant regulatory mechanism in HCC remains unclear. Therefore, we performed an RNA sequence to explore the underlying mechanism. Reports indicate that several lncRNAs work in conjunction with chromatin-modifying enzymes to either increase or decrease epigenetic target gene expression. Results from this study indicated that LINC01419 could bind to EZH2, a key component of methyltransferase PRC2.This suggested that LINC01419 may promote the development of HCC through transcriptional regulation of target genes. The genes are associated with HCC cell progression as they interact with EZH2. The oncogene, EZH2, plays a significant role in HCC by promoting cell proliferation and metastasis [12]. Besides, EZH2 can interact with lncRNAs to catalyze H3K27me3 in the promoter regions of the target gene, thereby mediating transcriptional silencing. In lncRNA-GATA6-AS1, for example, FZD4 expression is reduced by recruiting EZH and H3K27me3 to the FZD4 promoter region [13]. Again, LncRNA GAS5 is known to downregulate MMP9 by recruiting EZH2 in the MMP9 promoter region [14]. Similarly, from our study, LINC01419 transcriptionally regulated target proliferation and migration-related genes by binding to EZH2. This promoted HCC progression.

Among the target genes regulated by LINC01419, we focused on RECK because it is known to suppress tumors in various cancers, for example, HCC [15]. The RECK gene was first isolated from the v-Ki Ras-transformed NIH/3T3 cell line and identified as a transformation suppressor gene [16]. RECK has been used to regulate matrix metalloproteinases (MMPs), NOTCH-signaling, and WNT7-signaling [17]. Studies have reported that the RECK gene functions as a negative target for oncogenic signals. When RECK is downregulated in human cancers, transcription or epigenetic changes occur [18]. From our findings, LINC01419 decreased the RECK expression level via H3K27me3. Besides, histone methylation and DNA methylation have been reported to play a synergistic role in deactivating the expression of target genes. This study reported that the LINC01419-EZH2 complex transcriptionally decreases RECK expression by binding at its H3K27me3promoter.This contributes to HCC cell proliferation and metastasis. Furthermore, we confirmed thatLINC01419 and EZH2 interact with FUS, an oncogenesis-associated RBP involved in transcriptional regulation and RNA processing [19]. We reported that LINC01419 stabilizes EZH2-mRNA through a FUS-mediated mechanism, this is similar to our previous findings [20]. The lncRNAs can also function through the ceRNA network [21]. We did not explore whether LINC01419 can sponge microRNAs in HCC. This is one of the limitations to this study and, therefore, needs future in-depth exploration.

LINC01419 upregulation in HCC may be caused by several mechanisms. Transcriptional regulation, for example, majorly leads to abnormal expression of lncRNA [22]. Analysis of the upstream region of the LINC01419 locus revealed two putative binding sites for c-jun, which are involved in cell proliferation and metastasis [23]. Further experiments revealed that c-jun could positively regulate LINC01419 expression by directly binding to its promoter region. Similarly, c-jun was upregulated in HCC tissues and enhanced LINC01419 expression. We, therefore, proposed that c-jun-induced elevated LINC01419 expression contributes to HCC tumorigenesis.

In conclusion, this study reports that elevated LINC01419 expression level results in poor outcomes in HCC. LINC01419 potentially suppresses RECK expression epigenetically via EZH2.This, therefore, promotes HCC progression. LINC01419 upregulation is induced by the transcription activation of c-jun. These findings proved that LINC01419 could provide a theoretical basis for clinical diagnosis and treatment of hepatocellular carcinoma.

## MATERIALS AND METHODS

### HCC samples collection

HCC samples and paired non-cancerous specimens were obtained from the People's Hospital of Shanxi Province and Affiliated Cancer Hospital and Institute of Guangzhou Medical University. Tissues were immediately frozen in liquid nitrogen after hepatectomies and stored in a refrigerator at −80°C. The study was reviewed and approved by the Institutional Review Board of People's Hospital of Shanxi Province and Affiliated Cancer Hospital and Institute of Guangzhou Medical University.

### HCC cells culturing

HCC cell lines (HepG2, Huh7, and Bel7402) were maintained in our laboratory. Cells were cultured in 5% CO_2_ at 37 °C. The cells were maintained in RPMI 1640 supplemented with 10% FBS.

### Cell transfection

pcDNA3.1-RECK, pcDNA3.1-EZH2, and pcDNA3.1-LINC01419 were obtained from RiboBio (Guangzhou, China).siRNAs that targetLINC01419 were obtained from Genechem (Shanghai, China). Cells were transfected with oligonucleotides using Lipofectamine 2000 (Invitrogen, USA).

### Quantitative real-time PCR analysis

Total RNA was extracted from tissues or cells using TRIzol reagent (Invitrogen, CA, USA) following the manufacturer’s protocol. The PrimeScript RT reagent Kit (TaKara) was used during RNA reverse transcription. RT-PCR analyses were performed using the Gotaq® Green Master Mix (TaKara). The results were normalized with GAPDH. All the primer sequences used for RT-qPCR are listed in Supplementary Table 1.

### Colony formation and trans-well assays

For the colony formation assays, HCC cells were inoculated into 6-well plates. Two weeks later, the forming colonies were fixed with 4 % paraformaldehyde and stained with Giemsa. The colonies were counted.

Cell migration assay was performed as previously described [24].

### RNA-pulldown assays

The sense and antisense of full-length lncRNA- 01419 were labeled with biotin. The labeled RNAs were added into MgCl_2._ The mixture was added into cell lysates. Subsequently, these mixtures were incubated at 4 °C for 4 hours and then washed in lysis buffer. The precipitated proteins were separated by SDS-PAGE and subjected to western blot assay.

### RNA immunoprecipitation (RIP) assay

The Magna RIP RNA binding protein immunoprecipitation kit (Millipore, USA) was used for the RIP assay. The assay was performed following the manufacturer’s protocol.

### Chromatin immunoprecipitation (ChIP) assays

The ChIP assay kit (Millipore, MA, USA) was used for CHIP assay. The experiment was performed following the manufacturer’s instructions. The PCR products were visualized on an agarose gel.

### Western blotting

Total protein was extracted from cells using RIPA. The proteins were separated by SDS-PAGE and transferred to PVDF membranes. The PVDF membranes were blocked in 5 % fat-free milk and incubated with specific primary antibodies at 4 °C overnight. Subsequently, the membranes were incubated with the second antibody for one hour at room temperature. Finally, the protein was detected using an enhanced chemiluminescence system.

### Statistical analysis

Graph Pad Prism 5.0software and SPSS 13.0 were used for statistical analyses. The values were presented as the mean ± S.E.M. Analyses of different groups were performed using one-way ANOVA or two-tailed Student’s t-test. P < 0.05 was considered statistically significant.

## Supplementary Material

Supplementary Figures

Supplementary Table 1
