# Supplementary material for: LINC01419 facilitates hepatocellular carcinoma growth and metastasis through targeting EZH2-regulated RECK
Source: Aging (Albany NY). 2020 Jun 10;12(11):11071–84. doi: 10.18632/aging.103321 (PMC7346057; doi:10.18632/aging.103321)
Supplement: Supplementary Figures [file aging-12-103321-s002..pdf]

SUPPLEMENTARY FIGURES

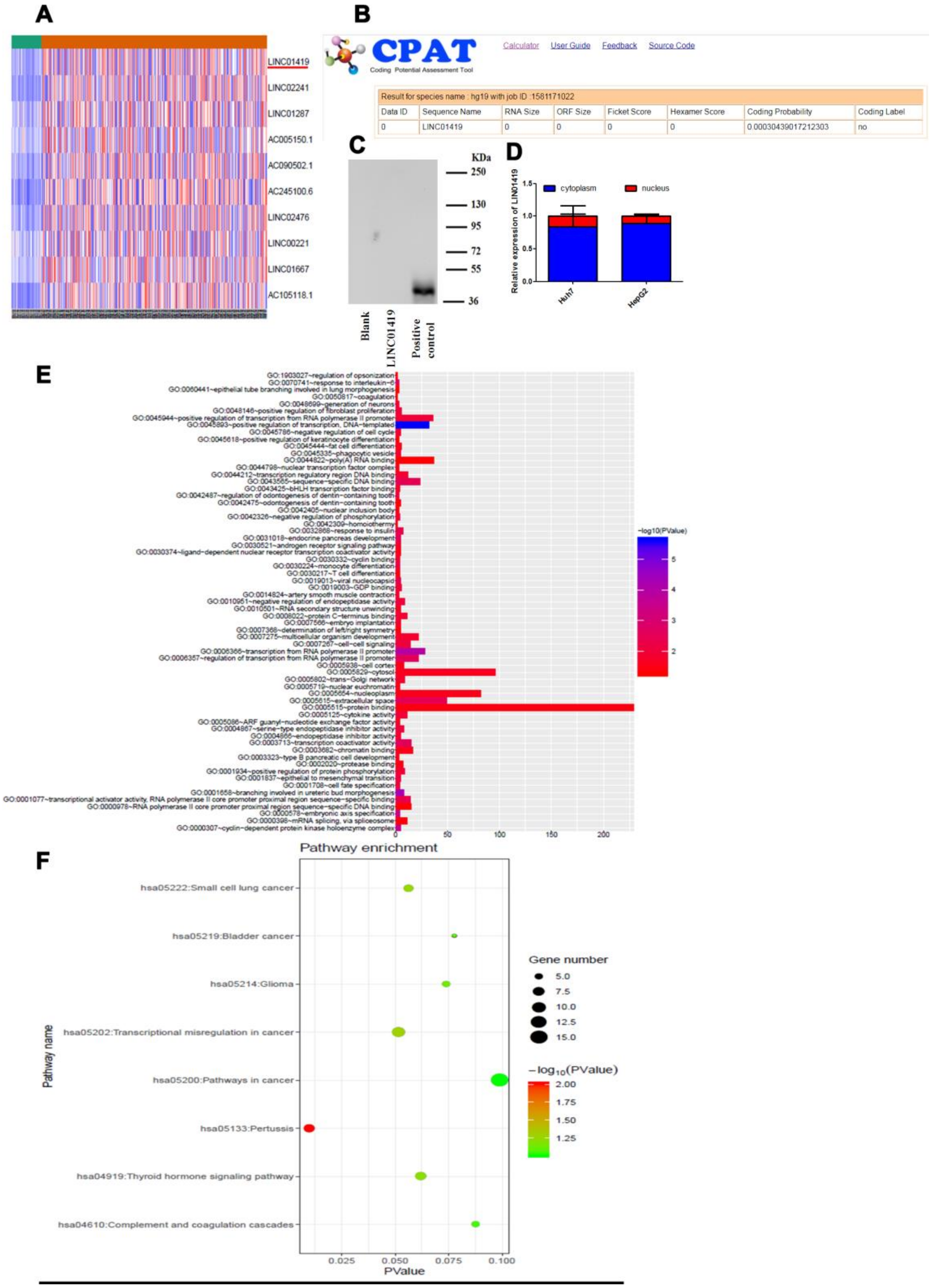

Supplementary Figure 1. (**A**) Hierarchical clustering analysis of lncRNAs differentially expressed (fold change > 2; p < 0.05) in HCC and normal tissues in the TCGA cohort. (**B**) Online bioinformatics analysis indicating protein LINC01419coding ability. (**C**) LINC01419 expression levels in cytoplasm and nucleus as examined by RT-PCR assay.(**D**) Subcellular fractionation and real-time PCR analysis showing that LINC01419 was mainly localized in the cytoplasm. (**E**) Gene ontology analysis for all genes with altered expression levels after LINC01419 down-regulation. (**F**) KEGG pathway enrichment for all genes with altered expression levels after LINC01419 down-regulation.

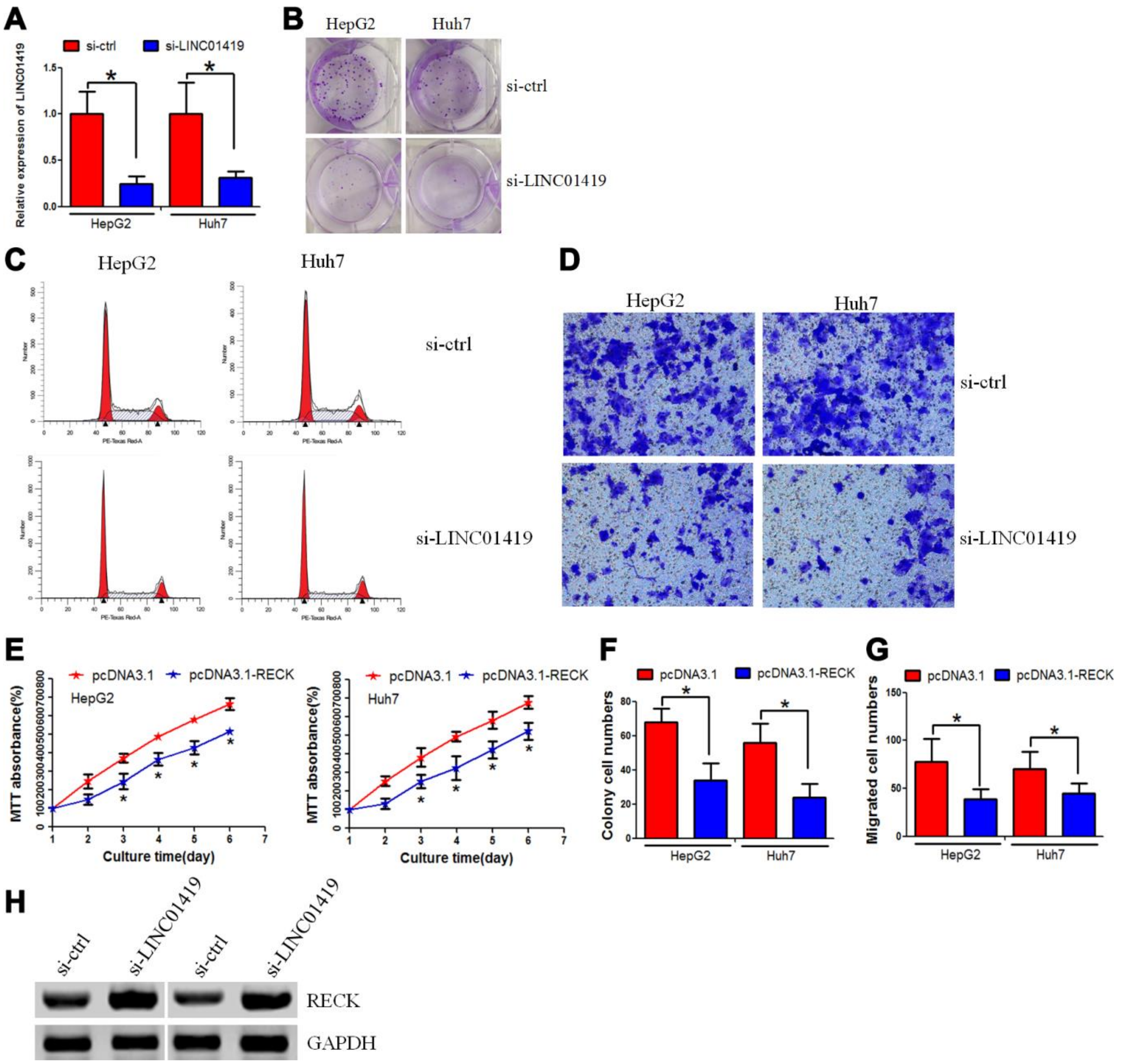

Supplementary Figure 2. (**A**) RT-PCR assay used toexamineLINC01419 knockdown efficiency in HCC cells. (**B**) The colony-forming ability impaired in LINC01419-silenced cells. (**C**) The cell cycle distribution measured using flow cytometry. (**D**) The HCC migration ability examined using transwell assays. (**E**) MTT assay revealed that overexpression of RECK decreased cell growth rate. (**F**) Colony formation assay revealed that overexpression of RECK impaired cell colony formation ability. (**G**) Transwell assay revealed that overexpression of RECK decreased cell migration ability. (**H**) Western blot assay revealed that inhibition of LINC01419 elevated RECK expression level.
