# Supplementary material for: LINC01419 facilitates hepatocellular carcinoma growth and metastasis through targeting EZH2-regulated RECK
Source: Aging (Albany NY). 2020 Jun 10;12(11):11071–84. doi: 10.18632/aging.103321 (PMC7346057; doi:10.18632/aging.103321)
Supplement: Supplementary Table 1 [file aging-12-103321-s001..pdf]

SUPPLEMENTARY TABLE

Supplementary Table 1. Primers used in the study.

	Forward primer 5'-3'	Reverse primer 5'-3'
RECK	GCGGGTGCATTGTGTTGTAA	TCTGCAAATAGCTGAGACGGT
EZH2	GGACTCAGAAGGCAGTGGAG	CTTGAGCTGTCTCAGTCGCA
LINC01419	TTTCTTGGCTCTCAGTGGCT	AACAGTCTCCCCTTTGTGATTT
GAPDH	AATGGGCAGCCGTTAGGAAA	GCGCCCAATACGACCAAATC
